# Supplementary material for: Lenticulostriate Vasculopathy in Very-Low-Birth-Weight Preterm Infants: A Longitudinal Cohort Study
Source: Children (Basel). 2021 Dec 9;8(12):1166. doi: 10.3390/children8121166 (PMC8700389; doi:10.3390/children8121166)
Supplement: Supplementary file 1 [file children-08-01166-s001.zip › children-1393375-supplementary.pdf]

Table S1. Multivariate analysis of the risk factors associated with late-onset lenticulostriate vasculopathy in very-low-birth-weight preterm neonates

	**Odds ratio**	**95% CI**	***p* value**
GA (increase 1 week)	1.294	0.890, 1.883	0.177
SGA	0.277	0.071, 1.084	0.065
Oxygen usage duration (increase 1 day)	1.030	1.003, 1.058	0.032

CI, confidence interval; GA, gestational age; SGA, small for gestational age
